# Minimally Invasive Controlled Growing Rods for the Surgical Treatment of Early-Onset Scoliosis—A Surgical Technique Video

**DOI:** 10.3390/jpm14060548

**Published:** 2024-05-21

**Authors:** Pawel Grabala

**Affiliations:** 1 Department of Pediatric Orthopedic Surgery and Traumatology, Medical University of Bialystok, Medical University of Bialystok Children’s Clinical Hospital, ul. Waszyngtona 17, 15-274 Bialystok, Poland; pgrabala@wp.pl; 2 Paley European Institute, Al. Rzeczypospolitej 1, 02-972 Warsaw, Poland; 3 Department of Neurosurgery with Department of Interventional Neurology, Medical University of Bialystok, Medical University of Bialystok Clinical Hospital, ul. M. Sklodowskiej-Curie 24A, 15-276 Balystok, Poland

**Keywords:** early-onset scoliosis, EOS, growing rod, minimally invasive controlled growing rods, MICGR

## Abstract

Background: Spinal deformities in children and adolescents can be easily divided into those occurring and diagnosed before the age of 10—early-onset scoliosis—and those occurring and diagnosed after the age of 10—late-onset scoliosis. When the curvature continues to progress and exceeds a Cobb angle of more than 60–65 degrees, surgical treatment should be considered. The most common treatment procedure for EOS is the surgical correction of the deformity using standard growing rods (SGRs), and in the case of congenital defects with additional hemivertebrae, it is the resection of the hemivertebra and short fusion. Minimally invasive controlled growing rods (MICGRs) need to be distracted every 6–9 months through a minimally invasive approach that involves sedation and neuromonitoring to obtain the best possible correction while minimizing complications. The aim of our study is to present a less-invasive surgical technique for MICGR implantation based on a two-case presentation—early-onset idiopathic scoliosis and congenital kyphosis. The surgical technique is the less-invasive percutaneous and subfascial implantation of MICGRs without long incisions in the back. Conclusions: The use of MICGRs is an alternative and safe surgical technique for patients undergoing surgical treatment for EOS. Without the risk of metallosis, like in other implant systems, and the need for replacement after 2 years of use, like in using magnetically controlled growing rods (MCGRs), the MICGR system can be used as a less-invasive procedure, allowing for the avoidance of many periodic invasive procedures in children with a wider opening of the spine (like in using standard growing rods), minimizing the number of planned hospitalizations, reducing the length of hospital stays, and reducing the physical and mental burdens on young patients, parents, and families.

## 1. Introduction

Spinal deformities in children and adolescents can be easily divided into those occurring and diagnosed before the age of 10—early-onset scoliosis—and those occurring and diagnosed after the age of 10—late-onset scoliosis [1,2,3]. Etiologically, these may be congenital, syndromic, neuromuscular, or idiopathic deformities [1,3,4]. Conservative treatment, rehabilitation, physiotherapy, and treatment with an orthopedic corset or brace are recommended [1,2,4,5]. When the curvature continues to progress and exceeds a Cobb angle of more than 60–65 degrees, surgical treatment should be considered [3,4,6]. It is known from data in the literature that the earlier the surgical treatment is performed, the more complications one can expect during the entire treatment process [7,8,9,10]. The most common treatment procedure for EOS is the surgical correction of the deformity using distraction-based systems, such as standard growing rods (SGRs), and in some cases of congenital defects with additional hemivertebrae, the treatment is the resection of the hemivertebra and short fusion [10,11]. When using standard growing rods, high rates of procedure-related complications are reported (up to 58%) [12]. The new generation of SGRs, magnetically controlled growing rods (MCGRs), also shows a high rate of complications, especially in relation to implant complications. According to the manufacturers’ recommendations, MCGR implants should be replaced after 2 years of use [13,14,15,16,17,18,19]. In 2023, an intermediate product between standard growing rods and magnetically controlled growing rods appeared. Minimally invasive controlled growing rods (MICGRs), which are without the potential hardware problems associated with MCGRs and SGRs, need distraction every 6–9 months through a minimally invasive approach that involves sedation and neuromonitoring to obtain the best possible correction effect while minimizing complications. Because this is a new product, and our center was one of the first in the world to introduce this technique, we decided to describe our surgical technique based on many years of experience in the implantation and use of growing-rod systems. The aim of our study is to present a less-invasive surgical technique for MICGR implantation based on example cases of children with early-onset idiopathic scoliosis and congenital scoliosis. Our surgical technique video is available to watch at Video S1: Minimally invasive controlled growing rods for the surgical treatment of early-onset scoliosis—a surgical technique video.

## 2. Example of Patients and the Surgical Technique

### 2.1. Surgical Technique

For all the operations, the patient was placed in a standard prone position using a Jackson surgical table. The neuromonitoring of the spinal cord is used for each spinal corrective surgery to reduce or even eliminate the risk of neurological deficits. Before each surgical procedure, we assess the structure of the vertebrae and the size of the pedicles and appropriately plan the selection of segmental screws that we will use during the surgery based on an MRI examination of the entire spine, which is routinely performed for all our patients who qualify for surgical treatment. The levels of stabilization are selected based on an analysis of radiological examinations of the patient in a standing position and after assessing the flexibility of the spine on bending films. The evaluation of the flexibility of each curvature was conducted on the bending films, with only the structural curvature undergoing stabilization, which was rectified through distraction employing growing rods with anchors positioned both above and below the said curvature. To control the fusion scope and minimize the spinal growth disruption for this purpose, the minimum required number of stabilization levels was restricted to 2 levels above and 2 levels below the curvature.

**Figure 1:**
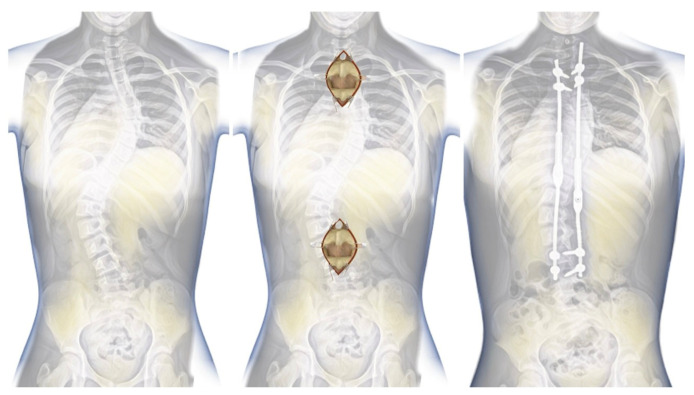
Less-invasive approach for the upper and lower spine.

Regardless of the size of the pedicles, we do not use screws smaller than 5.5 mm in diameter to obtain the best possible biomechanical properties during correction, excellent support points, and stability [20,21]; we have confirmed in several of our studies that this is safe and gives much better correction options for spinal deformation. Typically, in the lower section of the spine, where the pedicles are the widest, we use screws with a diameter of 6.5 mm or 6.0 mm, and in the upper thoracic section, we use screws with a diameter of 6.0 mm or 5.5 mm. If the pedicles are completely missing or dysplastic, we additionally place hooks at the highest level on the transverse processes to minimize the risk of pull-out and PJK. If the pedicles are missing or they are dysplastic, the screws are usually inserted using an extra-pedicular technique in accordance with the methods described in the literature [22,23]. The surgical technique for MICGRs is similar to the implantation of the SGR [4,10] and MCGR implantation techniques that we have previously described [21]. In our opinion, the most important thing is minimally invasive access to the spine and the exposure of only the levels at which screws and hooks will be implanted. In children, the greater and wider the access, the greater the risk of spontaneous fusion, which is not desired [16,17,24]. Although in adults, one can speak of a minimally invasive technique; it should be called a less-invasive technique because of the cuts that are performed. After positioning the patient under the control of the C-arm, the planned levels of screw implantation are marked on the skin with a marker; in most cases of early-onset scoliosis, these are levels T2–T4 and L1–L3. The next step is to make a skin incision that is about 4–5 cm long (depending on the child’s height) to access only the screw implantation levels (Figure 1).

**Figure 2:**
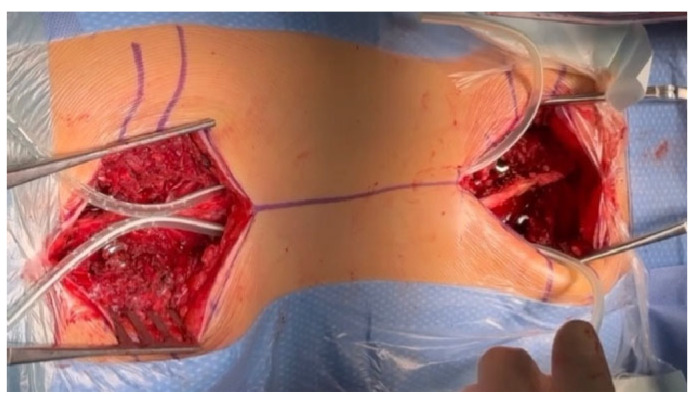
The subfascial tunneling for rod insertion. Drains were inserted into the resulting tunnels, after which a properly selected and cut growing rod was inserted.

From the point of view of spontaneous fusion [16,17,24], which is common in young children, it is extremely important to prepare the implant attachment points using two small, separate incisions, which are as small as possible, to insert the screws while leaving the rest of the spine intact. We implant screws using the free-hand technique [23], but other techniques can also be used with support from a C-arm, an O-arm, navigation, or robotics. After checking the correct position of the C-arm screws and neuromonitoring, we make two subfascial tunnels on the left and right sides of the spine to insert the growing rods. The tunnels should be created subfascially and should avoid entering the muscles, which may also increase the risk of autofusion at the top of the curve [15,24]. Using an appropriately sized pair of paean forceps, we produce rod tunnels directly under the fascia, not reaching the spine. To facilitate the guidance of the rods without damaging the muscles and to avoid pleural perforation, No. 16 drains are inserted into the resulting tunnels, after which a properly selected and cut growing rod is inserted (as shown in Figure 2).

**Figure 3:**
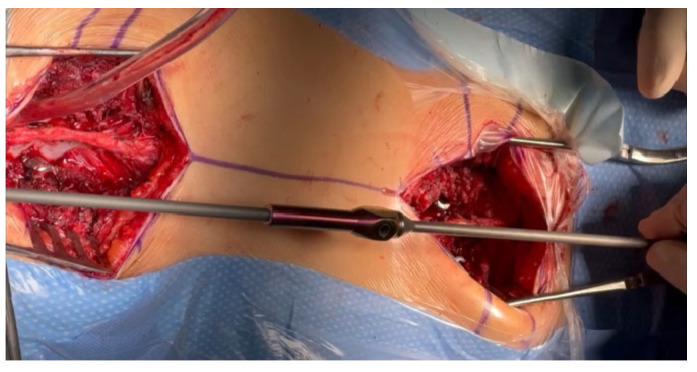
The measurement of the MICGR.

First, we apply the rod to the stabilization points to determine its length and determine the best method for obtaining the best bending of the rod (Figure 3). The next step is properly cutting and bending the rods.

**Figure 4:**
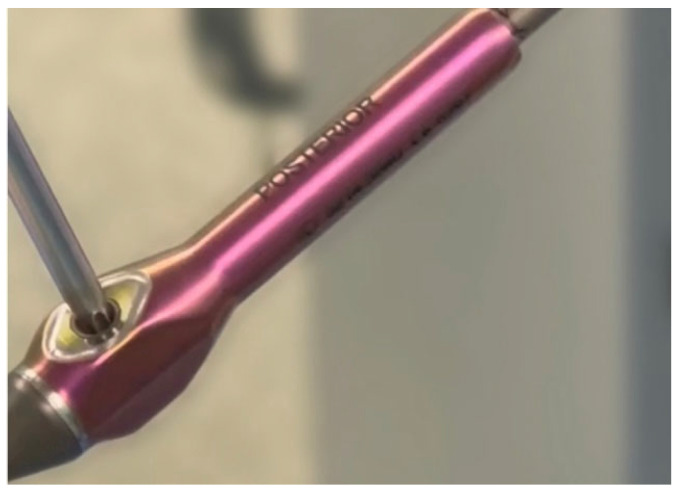
The actuator test should be performed before bending the rods and after they have been properly contoured.

After measuring and cutting the MICGR, we need to test the effectiveness of the rod-expanding actuator. The actuator test should be performed before bending the rods and after they have been properly contoured for the restoration of the sagittal balance to exclude the possibility of damage to the mechanism while bending the rod (Figure 4).

**Figure 5:**
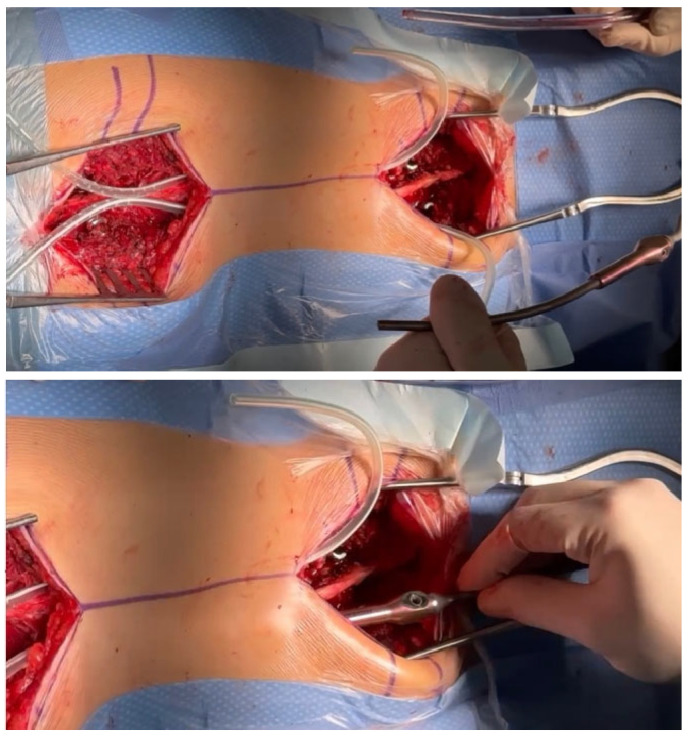
We passed the rod through a previously prepared tunnel, placing the end of the tubing at the end of the rod.

A very important element of bending the rods is modeling them 1 cm above and below the ends of the actuator. This is to prevent mechanical damage to the actuator. However, this method is not perfect, and because of the impossibility for bending the actuator in any way, we must deal with a completely straight part of the rod (70 mm or 90 mm long), depending on the type of rod that is used. Another important element is the planning of the implantation of MICGRs in such a way that the rod actuators are at the same height. When the rods are properly prepared, we can start inserting them. Our technique is to place the first rod on the left side (concave) for right-hand curves and vice versa for left-hand curves. We pass the rod through a previously prepared tunnel, placing the end of the tubing at the end of the rod (Figure 5).

**Figure 6:**
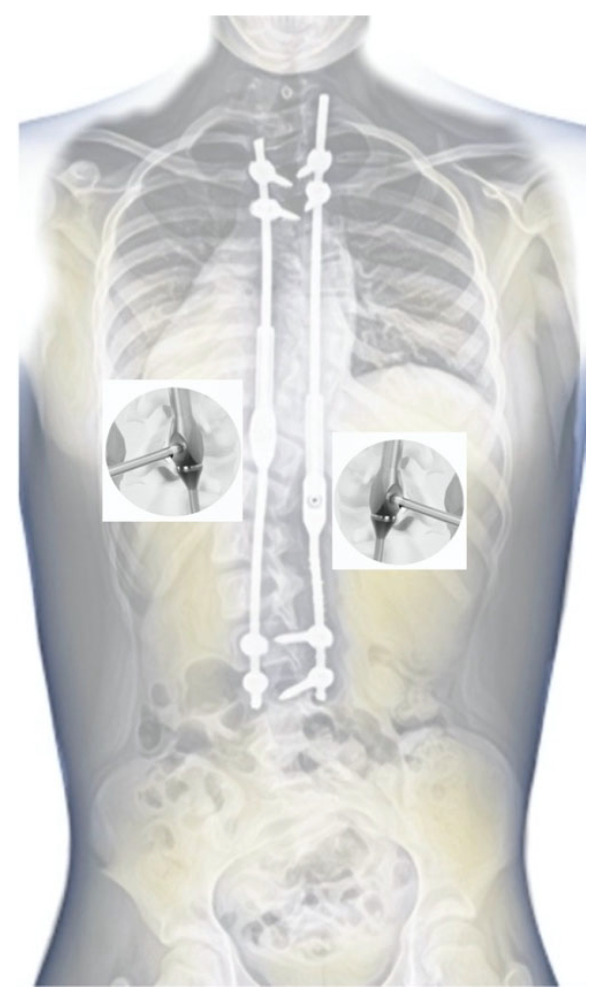
We distracted the MICGRs every 6–9 months through the minimally invasive approach with 1 cm while using sedation and neuromonitoring to obtain the best possible correction effect while minimizing complications.

We guide the rod cephalad. After securing the rod to the screw heads, we de-rotate it to the appropriate sagittal position and temporarily lock it. We then repeat the process on the opposite side with the second rod. After the rod insertion, we perform gentle distraction distally across the base on the proximally set screws and complete a final tightening. We do not routinely use a proximal cross-connector between the rods. In patients with poor bone quality or missing pedicles at the top of the structure, when inserting juxtapedicular screws [22], we use transverse process hooks at the top to prevent the upper implant from breaking out. We then complete the final irrigation, bone decortication at the screws, coating of the bone grafts with vancomycin powder, and standard closure. During the surgery, we use safety distraction for the neuromonitoring of the spinal cord. There were no changes in SSEP and MEEP. There were no postoperative complications. All the patients were discharged on the sixth day, with an orthopedic corset (for three months) until solid fusion is achieved at the segmental screw implantation levels to reduce the risk of screw pull-out. We distracted the MICGRs every 6–9 months because of the growing potential of the child to obtain the best possible correction effect while minimizing complications through the minimally invasive approach using sedation and neuromonitoring (Figure 6). Our surgical technique video is available to watch at Video S1: Minimally invasive controlled growing rods for the surgical treatment of early-onset scoliosis—a surgical technique video.

### 2.2. Example of Early-Onset Idiopathic Scoliosis

**Figure 7:**
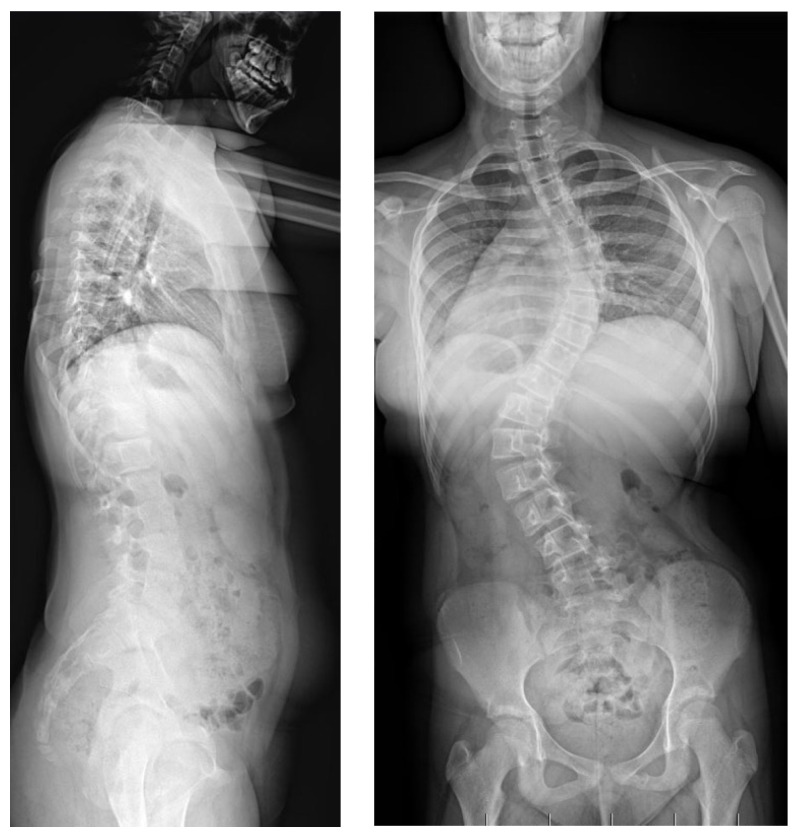
Preoperative images in a standing position: (from (**left**) to (**right**)) LAT and AP X-rays at 9 years old (76 degrees for the main curve).

A 9-year-old girl was admitted to our clinic with rapidly progressing thoracic scoliosis. Previously, the patient had undergone conservative treatment with a brace (23 hours per day) and standard physical therapy for early-onset scoliosis treatment. The patient had no other disorders. The major curve of the thoracic spine’s Cobb angle was 30  degrees at 7 years of age, 50 degrees at 8 years of age, and 74 degrees at 9 years of age, when she qualified for surgical treatment in our department (Figure 7).

**Figure 8:**
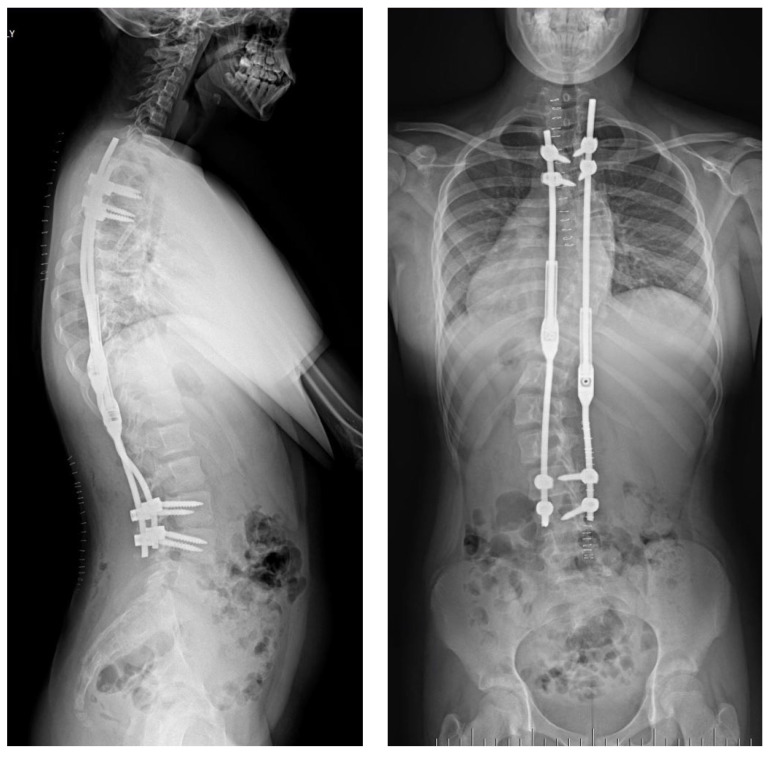
Standing postoperative X-rays (LAT and AP).

The spinal cord was intact in the MRI examination of the whole spine. No other congenital deformities were detected. The patient was born through natural childbirth without any comorbidities. All the examinations, specialist consultations, and clinical statuses showed other causes of the spinal deformity. We diagnosed early-onset idiopathic scoliosis, which qualified for surgical treatment with growing rods that were inserted via a less-invasive approach with subfascial rod placement (Figure 8).

### 2.3. Example of Congenital Kyphosis

**Figure 9:**
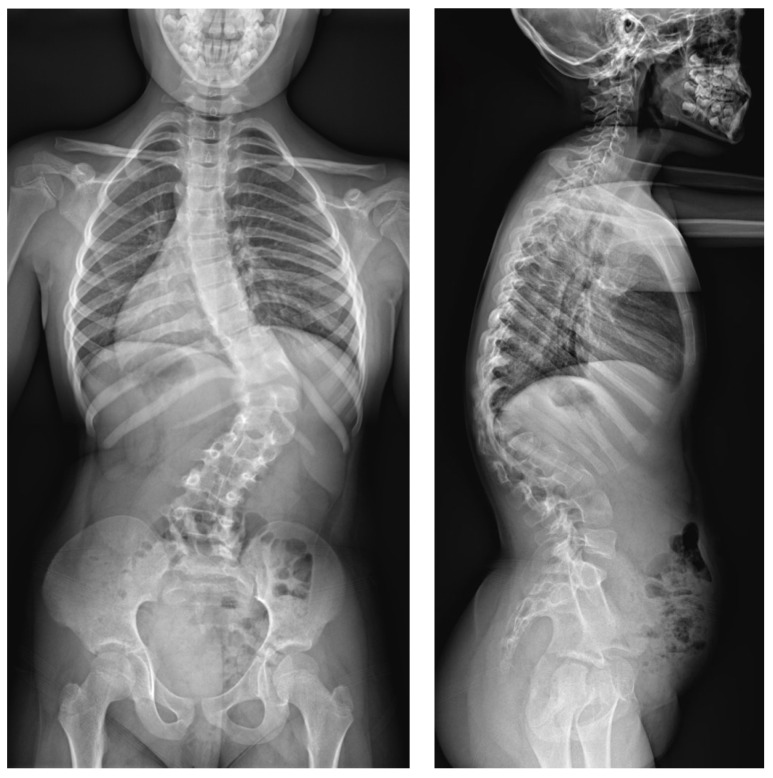
Preoperative images in a standing position: (from (**left**) to (**right**)) AP and LAT X-rays.

A 6-year-old girl was admitted to our clinic with rapidly progressing thoracolumbar kyphosis. Previously, the patient had undergone conservative treatment with a brace (23 hours per day) and physical therapy at another clinic. The treatment course did not provide the expected results, and the progression of focal kyphosis was not stopped. Magnetic resonance imaging was performed. The examination did not find any other congenital deformities, except for the wedge-shaped structure of the L1 vertebra. The malformation of the L1 vertebra and its subsequent wedging led to the advancement of the curvature in the sagittal profile as the individual underwent growth. The major curve of the thoracic spine’s Cobb angle was 67 degrees, and the thoracic focal kyphosis was 82 degrees at 6 years of age (Figure 9). The patient was born through natural childbirth without any comorbidities.

**Figure 10:**
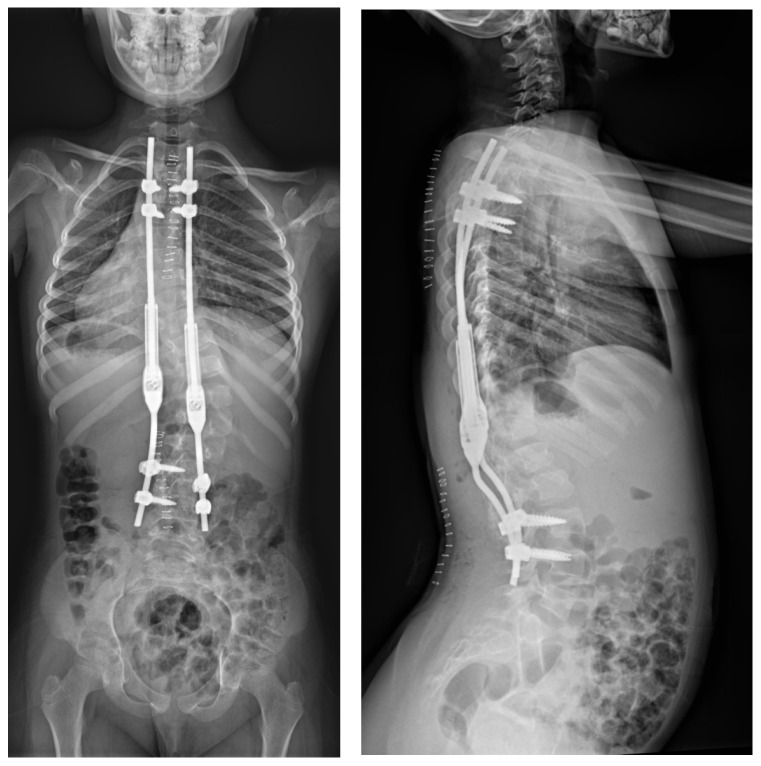
Standing postoperative X-rays (LAT and AP).

All the examinations, specialist consultations, and clinical statuses showed other causes of the spinal deformity. We diagnosed congenital kyphosis, and the child qualified for surgical treatment with growing rods, which were inserted via a less-invasive approach with subfascial rod placement (Figure 10).

## 3. Discussion

The treatment of early-onset scoliosis consists of an initial phase of observation and rehabilitation. If the curvature progresses, it is recommended to use a series of casts and an orthopedic corset or brace [5,25]. Congenital curvatures are not susceptible to conservative treatment and require surgical treatment in the case of the proven progression of the curvature [1,4,5,25]. If there are no effects of the conservative treatment and further progression of the spinal curvature exceeding a Cobb angle of 60–65 degrees, surgical treatment should be considered [1,3,4]. We have several options to choose from, such as SGRs, MCGRs, MICGRs, and vertical expandable prosthetic titanium ribs (VEPTRs); growth guidance systems, like Shilla; the Luque trolley technique; or newer concepts, like spring distraction systems for the dynamic growth guidance of early-onset scoliosis or vertebral body tethering [1,9,26,27,28,29,30,31,32,33,34,35,36]. The most appropriate technique should be selected for the curve and the patient. According to our many years of experience, to minimize complications, the procedure should be as minimally invasive as possible and performed as late as possible, i.e., surgical treatment should be postponed as long as possible because of the complication rate [8,9,10,15,16]. Growing rods, inserted through a less-invasive incision, correct the spinal curvature by initially distracting the spine; they then maintain the resulting correction of the deformity by controlling both the spinal curvature and growth and promoting growth with further non-invasive extensions of the internal fixator. The less-invasive option for extending MICGRs reduces the risk of complications and significantly shortens the length of hospitalizations, providing the most optimal control over the curvature in addition to the maximum stretching to allow for growth and safety because the lengthening of the spine and instrumentation is controlled using the neuromonitoring of the spinal cord through minimally invasive access using a skin incision of less than approximately 1 cm over the lengthening mechanism [36,37,38,39,40]. The MICGR system might seem to be ideal, but it cannot be used for every patient with EOS, and the treatment with this system is limited. Not every patient can be implanted with this type of rod because of the size and the limited possibilities for intraoperative bending. More precisely, these rods do not fit all curvatures and patients. There is also a risk of complications in this system, as in any other. The available literature has shown some defects and complications during treatment with SGRs, MCGRs, and GGSs, such as deep wound infection, kyphosis of the proximal junctions (PJK), breakage of rods, inability to distract the instruments, and self-healing of the spine before the end of the treatment [13,37,39,40]. According to data available in the literature, implant failure is most often associated with the breakage of the magnetic rod or loss of the ability to perform further distraction [13,40]. In patients treated with the MCGR system, in medium- and long-term follow-ups, the total number of complications was from approximately 35% to 73%, but this was also related to the results of the treatment of scoliosis of various etiologies because, as we know, scoliosis with an etiology other than idiopathic is associated with an increased risk of overall complications [7,8,13,14,15,16,17,24,39,40,41,42]. Compared with other surgical techniques used to treat EOS, MCGRs can be introduced as a less-invasive surgical technique using a mini-open approach [26,27,28,29,30,31,32,33,34,35,36]. The implantation of the new MICGR system is also similar [21]. To reduce the high risk of spontaneous spinal fusion in the growing spine [10,43,44], during surgical procedures with wide access to the spine, MCGRs should be placed subfascially. Metallosis resulting from the use of MCGRs appears to be of significant clinical importance, and, therefore, the product registration requires the replacement of MCGR rods 2 years after the primary implantation, which is not the case with MICGRs [7,8,14,15,16,17,24]. Replacement every 2 years increases the number of surgeries that are performed, which may increase the risks of spontaneous spondylodesis and infection [7,8,9,14,15,16,17,24]. The use of MICGRs is a new alternative to both SGRs and MCGRs. By eliminating metallosis and the need for a wide opening for the instrument distraction, MICGRs are an interesting and promising alternative—a safe and effective surgical technique in patients undergoing surgical treatment for EOS in comparison with other techniques [1,3,4,6,7,9,11,13,19,21,23].

## 4. Conclusions

The use of MICGRs is an alternative and safe surgical technique for patients undergoing surgical treatment for EOS. Without the risk of metallosis, like in other implant systems, and the need for replacement after 2 years of use, like in using magnetically controlled growing rods (MCGRs), the MICGR system can be used as a less-invasive procedure, allowing for the avoidance of many periodic invasive procedures in children with a wider opening of the spine (like in using standard growing rods), minimizing the number of planned hospitalizations, reducing the length of hospital stays, and reducing the physical and mental burdens on young patients, parents, and families.

## Data Availability

Not applicable.

## Supplementary Materials

The following supporting information can be downloaded at https://www.mdpi.com/article/10.3390/jpm14060548/s1: Video S1: Minimally invasive controlled growing rods for the surgical treatment of early-onset scoliosis—a surgical technique video.
